# Entropy Generation in 2D Lid-Driven Porous Container with the Presence of Obstacles of Different Shapes and under the Influences of Buoyancy and Lorentz Forces

**DOI:** 10.3390/nano12132206

**Published:** 2022-06-27

**Authors:** Apichit Maneengam, Houssem Laidoudi, Aissa Abderrahmane, Ghulam Rasool, Kamel Guedri, Wajaree Weera, Obai Younis, Belgacem Bouallegue

**Affiliations:** 1 Department of Mechanical Engineering Technology, College of Industrial Technology, King Mongkut’s University of Technology North Bangkok, Bangkok 10800, Thailand; apichit.m@cit.kmutnb.ac.th; 2 Laborarory of Sciences and Marine Engineering (LSIM), Faculty of Mechanical Engineering, USTO-MB, El-Menaouer, Oran 31000, Algeria; laidoudi@univ-usto.dz; 3 Laboratoire de Physique Quantique de la Matière et Modélisation Mathématique (LPQ3M), University of Mascara, Mascara 29000, Algeria; a.aissa@univ-mascara.dz; 4 Institute of Intelligent Machinery, Faculty of Materials and Manufacturing, Beijing University of Technology, Beijing 100124, China; grasool@zju.edu.cn; 5 Mechanical Engineering Department, College of Engineering and Islamic Architecture, Umm Al-Qura University, P.O. Box 5555, Makkah 21955, Saudi Arabia; kmguedri@uqu.edu.sa; 6 Department of Mathematics, Faculty of Science, Khon Kaen University, Khon Kaen 40002, Thailand; 7 Department of Mechanical Engineering, Prince Sattam Bin Abdulaziz University, Wadi Addwaser 11991, Saudi Arabia; oubeytaha@hotmail.com; 8 College of Computer Science, King Khalid University, Abha 61413, Saudi Arabia; bbelgacem@kku.edu.sa; 9 Electronics and Micro-Electronics Laboratory, Faculty of Sciences of Monastir, University of Monastir, Monastir 1002, Tunisia

**Keywords:** entropy generation, mixed convection, hybrid nanofluids, steady-state, heat transfer

## Abstract

This paper includes a numerical investigation of a hybrid fluid containing 4% of Al_2_O_3_-Cu nanoparticles in a lid-driven container. The upper wall of the container has a high temperature and is movable. The lower wall is cool and wavy. An obstacle is set in the middle of the container for its effect on thermal activity. The medium is permeable to the fluid, and the entire system is immersed in a fixed-effect magnetic field. The digital simulation is achieved using the technique of Galerkin finite element (GFEM) which solves the differential equations. This investigation aims to know the pattern of heat transfer between the lateral walls and the lower wall of the container through the intervention of a set of conditions and criteria, namely: the strength of the magnetic field changes in the range of (Ha = 0 to 100); the chamber porosity varies in the range of (Da = 10^−5^ to 10^−2^); the strength of buoyancy force is varied according to the Grashof number (Gr = 10^2^ to 10^4^); the cross-section of the baffle includes the following shapes—elliptical, square, triangular and circular; the surface of the lower wall contains waves; and the number changes (N = 2 to 8). Through this research, it was concluded that the triangular shape of the baffle is the best in terms of thermal activity. Also, increasing the number of lower-wall waves reduces thermal activity. For example, the change in the shape of the obstacle from the elliptical to triangular raises the value of Nu number at a rate of 15.54% for Ha = 0, N = 8, and Gr = 10^4^.

## 1. Introduction

Thermal transfer in enclosed rooms remains one of the most important topics in recent research due to its wide-ranging uses in many engineering applications. Among the most important of these applications are the following: cooling systems in electronic devices, building air conditioning and ventilation systems, heat exchangers, solar collators, nuclear reactors, and so on. Studies on this research type are conducted through real experiments [1,2,3] or numerical simulations [4,5,6] using very advanced mathematical methods. Most recent studies adopt the second type due to its speed in reaching a solution, as well as its low cost. The results of these studies allow us to understand the thermal activity as well as to obtain some coefficients needed in calculating the heat flux.

A lid-driven cavity is a technical term for a completely enclosed chamber containing a fluid in which one of its walls undergoes horizontal motion [7,8,9,10]. Several studies have been carried out on this subject for the purpose of presenting the elements that contribute to raising the heat transfer between the internally retained fluid and the chamber walls [11,12,13,14,15]. In general, it is understood that thermal transfer is related to two main issues, namely, the fluid’s thermal proprieties and the chamber’s geometrical shape.

In this part, we review the most important recent research and the results that have been extracted to understand what was previously examined. Selimefendigil [16] studied the lid-driven of the ordinary chamber. In the center of the room, there is an obstacle of circular and elliptical cross-sections. The fluid used here is simple water to which particulate elements of solid bodies have been added. It was discovered that microparticles added to the water improve heat transfer by up to 120%. Ghasemi and Siavashi [17] used the numerical method of lattice Boltzmann for the purpose of studying mixed convection of nanofluid in a 3D room under the presence of the magnetohydrodynamic (MHD) effect. The room walls are all straight, and there are no obstructions inside the container. The study showed the possibility of a decrease in heat transfer by up to 30%. Alsabery et al. [18] combined in their work a group of influences aimed at increasing the efficiency of the flow in heat transfer. These elements are: fixing an obstacle in the center of the chamber, using the small particles to strengthen the thermal proprieties of the fluid, and, finally, making some geometrical changes on the walls of the container. It was found that the small particle size strengthens the energy transfer of the fluid. Gibanov et al. [19] studied the thermal activity in a lid-driven room of a square cross-section. In their work, the chamber’s lower half is considered porous, while the upper half is simple. The results showed that the heat transfer, in this case, is affected by a number of factors. Gangawane et al. [20] placed a triangular obstacle in the middle of the lid-drive room. They then changed its place to determine the effective location that allows for increasing thermal activity. Gangawane and Manikandan [21] also tested working on the triangular shape of the obstacle, but this time the walls of the obstacle are not thermally insulated but rather have a high temperature. This work aimed to understand the process of cooling the obstacle by fluid movement through the movable upper wall of the space. Alsabery et al. [22] conducted a study on the effect of obstacle diameter on the dynamic comportment of the fluid and its retroactive effect on thermal activity. Alsabery et al. [23] incorporated the effect of the cylinder’s diameter and changed the shape of the chamber wall from regular to zigzag. 

In fact, there are still a large number of recent researches that are closely related to the topic [24,25,26,27,28,29,30,31,32,33,34,35,36,37,38,39,40]. In a nutshell, all these researches aim to find the most useful way to improve the heat transfer within the lid-driven room. To achieve this, some researchers resorted to improving the proprieties of the ordinary fluid by adding some very fine solid particles. Others changed the geometric shape of the room walls, i.e., from a simple wall to a wall containing zigzags and ribs. Also, a team of researchers has included some obstacles inside the room. Finally, a class applied some external forces to the system, such as the effect of the magnetic field. 

Through our observation of the previous research, we wanted to address a study that combines all of the above because of, as noted, the absence of work that combines the effect of all the porosity of the space and the presence of an obstacle inside the room in different forms for a wavy wall of the chamber and under the influence of both thermal buoyancy and Lorentz forces. In addition, there are works focused on using nanofluids to enhance thermal proprieties [41,42,43,44,45,46,47,48,49,50,51,52,53,54,55,56]. These works witness the combination of nanofluids’ thermal proprieties and a magnetic field’s effect (magnetohydrodynamics). 

In this part, we review the most important recent research and the results that have been extracted to understand what was previously examined. Selimefendigil [16] studied the lid-driven ordinary chamber. In the center of the room, there is an obstacle of circular and elliptical cross-sections. Based on what we saw in the previous research, we wanted to achieve a study that combined all of the above. This is because there has not been any work that combines the effects of all the holes in the space and the presence of an obstacle inside the room in different ways for a wavy wall of the chamber and under the influence of both thermal buoyancy and Lorentz forces.

As a result, this work is about a lid-driven room with a square cross-section, with a thermally insulated obstacle of various cross-sections (elliptical, square, triangular, and circular) in the middle. The bottom wall of the container is wavy and has an elevated temperature, and the lateral sides have a low temperature. The chamber contains water with 4% of nanoparticles, and it has permeability while the system is under the magnetic field’s influence. The work aims to show how the nanofluid transfers the thermal energy from the hot wall towards the cold sides under the impact of the studied elements.

This work examines some points that were not touched upon before, which are:

- Studying the porosity of the medium by changing the value of the Darcy number (Da);
- Changing the shape of the cross-section of the obstacle of the lid-driven room;
- A change in the strength of the magnetic field, according to the value of the Hartmann number;
- A change in the value of the Grashof number and a change in the effect of thermal buoyancy strength;
- The impact of the wave number of the lower wall;
- Knowing the conditions that allow the strengthening of heat transfer; and
- Identify areas where the flow is stopped (stagnated), in order to avoid.

The results of this research expand the understanding and support the results of previous works. Also, some value of these results can be extracted for use in engineering applications. Indeed, this type of research can be applied in many engineering fields, including cooling systems in electronic devices, special thermal exchangers of limited use, medical drug manufacturing, and others.

## 2. Physical Model and Appropriate Mathematical Models

**Figure 1:**
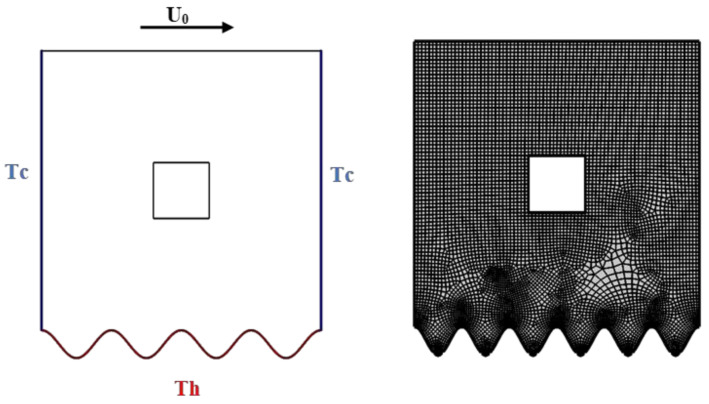
Physical domain and mesh.

A permeable medium is considered a defined mechanism. Consider the steady 2D, forced convection flow of water-driven Al_2_O_3_-Cu nanoparticles enclosed in a wavy chamber. The upper surface (Y = 1) of the cavity is divided into parts (0 ≤ X ≤ 1 and 0.5 ≤ X ≤ 1) that are moving with a velocity of U_0_. The lower wavy wall of the cavity is heated with temperature (T = T_H_), and the remaining two sides are kept cold (T = T_C_) with zero velocities. A differently shaped (elliptic, cylindrical, square, or triangular) obstacle is placed inside. The configuration of a physical model is presented in Figure 1.

## 3. The Governing Equations

The equations that govern the physical problem under consideration (Equations (2)–(4)) are the continuity, momentum, and energy equations [53]. These equations read:

(1) $$\frac{\partial u}{\partial \;x}+\frac{\partial v}{\partial \;y}=0$$

(2) $$\rho_{nf}\left(u\frac{\partial \;u}{\partial \;x}+v\frac{\partial u}{\partial \;y}\right)=-\frac{\partial \;P}{\partial \;x}+\mu_{nf}\left(\frac{\partial^{2}u}{\partial \;x^{2}}+\frac{\partial^{2}u}{\partial \;y^{2}}\right)-\frac{v_{nf}}{K}u-\frac{C_{F}}{\sqrt{K}}\left|\vec{v}\right|u$$

(3) $$\;\rho_{nf}\left(u\frac{\partial v}{\partial x}+v\frac{\partial v}{\partial y}\right)=-\frac{\partial P}{\partial y}+\mu_{nf}\left(\frac{\partial^{2}v}{\partial x^{2}}+\frac{\partial^{2}v}{\partial y^{2}}\right)-\frac{v_{nf}}{K}\nu -\frac{C_{F}}{\sqrt{K}}\left|\vec{v}\right|\nu \;\;+\rho_{nf}\beta_{nf}g\;(T-T_{c})-\sigma_{nf}B^{2}v$$

(4) $${\left(\rho C_{p}\right)}_{\;nf}\left(u\frac{\partial \;T}{\partial \;x}+v\frac{\partial T}{\partial \;y}\right)=k_{nf}\left(\frac{\partial^{2}T}{\partial \;x^{2}}+\frac{\partial^{2}T}{\partial \;y^{2}}\right)$$

where $\vec{V},\varepsilon ,K$, and $C_{F}$ are the velocity vector, the porosity, the permeability, and the Forchheimer coefficient, respectively. Also, g, $\rho_{nf},$ and $\beta_{\mathrm{af}}$ are the gravity, the density, and the thermal expansion coefficient, respectively.

The following thermophysical characteristics were employed in the current work:

(5) $$\mathrm{density}\;\rho_{nf}=\left(1-\varphi \right)\rho_{f}+\varphi \rho_{P}$$

(6) $$\mathrm{heat}\;\mathrm{capacity}\;{\left(\rho C_{p}\right)}_{nf}=\left(1-\varphi \right){\left(\rho C_{p}\right)}_{bf}+\varphi {\left(\rho C_{p}\right)}_{P}$$

(7) $$\mathrm{thermal}\;\mathrm{expansion}\;\mathrm{coefficient}\;{\left(\rho \beta \right)}_{nf}=\left(1-\varphi \right){\left(\rho \beta \right)}_{f}+\varphi {\left(\rho \beta \right)}_{P}$$

(8) $$\mathrm{electrical}\;\mathrm{conductivity}\;{\left(\sigma \right)}_{nf}=\left(1-\varphi \right){\left(\sigma \right)}_{f}+\varphi {\left(\sigma \right)}_{P}$$

(9) $$\mathrm{thermal}\;\mathrm{conductivity}\;k_{nf}=k_{bf}\left(4.97\;\varphi^{2}+2.72\;\varphi +1\right)$$

(10) $$\mathrm{dynamic}\;\mathrm{viscosity}\;\mu_{nf}=\mu_{bf}\left(123\;\varphi^{2}+7.3\;\varphi +1\right)$$

Equations (5)–(10) [51] make use of the constants given in Table 1.

Dimensionless forms of the governing equations were obtained by using cavity length L and lid-driven velocity U_0_:

(11) $$X=\frac{x}{L},\;Y=\frac{y}{L},\;\;U=\frac{u}{U_{0}}\;,V=\frac{v}{U_{0}}\;\theta =\frac{T-T_{C}}{T_{h}-T_{C}},\;\;\theta_{s}=\frac{T_{s}-T_{C}}{T_{h}-T_{C}}\;\;P=\frac{p}{\rho_{nf}U_{0}^{2}},\;Pr=\frac{v_{f}}{a_{f}},\;Ri=\frac{Gr}{Re^{2}},\;$$

The Richardson number is given by:

Ri = Gr/Re^2^

Then, the non-dimensional governing equations can be written as:

(12) $$\frac{\partial U}{\partial X}+\frac{\partial V}{\partial Y}=0$$

(13) $$U\frac{\partial U}{\partial X}+V\frac{\partial U}{\partial Y}=-\frac{\partial P}{\partial X}+\frac{1}{Re}\frac{\mu_{\mathrm{hnf}}}{\mu_{f}}\frac{\rho_{f}}{\rho_{\mathrm{hnf}}}\left(\frac{\partial^{2}U}{\partial X^{2}}+\frac{\partial^{2}U}{\partial Y^{2}}\right)-\frac{\mathrm{Pr}}{\mathrm{Da}}U-\frac{C_{F}}{\sqrt{\mathrm{Da}}}\left|\vec{V}\right|U$$

(14) $$\begin{array}{cc} U\frac{\partial V}{\partial X}+V\frac{\partial V}{\partial Y}= & -\frac{\partial P}{\partial Y}+\frac{1}{Re}\frac{\mu_{\mathrm{hnf}}}{\mu_{f}}\frac{\rho_{f}}{\rho_{\mathrm{hnf}}}\left(\frac{\partial^{2}V}{\partial X^{2}}+\frac{\partial^{2}V}{\partial Y^{2}}\right)-\frac{\mathrm{Pr}}{\mathrm{Da}}V-\frac{C_{F}}{\sqrt{\mathrm{Da}}}\left|\vec{V}\right|V\\ & +\frac{{\left(\rho \beta \right)}_{\mathrm{hnf}}}{\rho_{\mathrm{hnf}}\beta_{f}}\mathrm{Ri}\theta -\frac{\sigma_{nf}{\mathrm{Ha}}^{2}V}{\rho_{nf}} \end{array}$$

(15) $$U\frac{\partial \theta }{\partial X}+V\frac{\partial \theta }{\partial Y}=\frac{\alpha_{\mathrm{hnf}}}{\alpha_{f}}\frac{1}{\mathrm{PrRe}}\left(\frac{\partial^{2}\theta }{\partial X^{2}}+\frac{\partial^{2}\theta }{\partial Y^{2}}\right)$$

The non-dimensional form of boundary conditions associated with Equations (12) and (15) read:

For the lower wavy hot wall:

(16) U=V=0, θ=1, 0≤X≤ 1, Y=0

For the cold upper wall:

(17) U=1, V=0, θ=0, 0≤X≤ 1, Y=1

For the left and right walls:

(18) $$U=V=0,\frac{\partial \theta }{\partial X}=0\;$$

at the concentric surface,

(19) $$U=V=0,\;\frac{\partial \theta }{\partial n}=0$$

The local Nusselt number calculated at the lower wavy hot wall reads:

(20) $$Nu_{s}=-\frac{k_{nf}}{k_{f}}{\left(\frac{\partial \theta }{\partial Y}\right)}_{Y=0}$$

The average Nusselt reads:

(21) $${\bar{Nu}}_{nf}=\int_{0}^{1}Nu_{s}dY$$

The entropy production reads [29]:

(22) $$S=\frac{k_{nf}}{T_{0}^{2}}\left[{\left(\frac{\partial T}{\partial x}\right)}^{2}+{\left(\frac{\partial T}{\partial y}\right)}^{2}\right]+\frac{\mu_{nf}}{T_{0}}\left[2\left({\left(\frac{\partial u}{\partial x}\right)}^{2}+{\left(\frac{\partial v}{\partial y}\right)}^{2}\right)+{\left(\frac{\partial u}{\partial x}+\frac{\partial v}{\partial x}\right)}^{2}\right]$$

Local entropy production non-dimensional form reads:

(23) $$S_{GEN}=\frac{k_{nf}}{k_{f}}\left[{\left(\frac{\partial \theta }{\partial X}\right)}^{2}+{\left(\frac{\partial \theta }{\partial Y}\right)}^{2}\right]+\frac{\mu_{nf}}{\mu_{f}}N_{\mu }\left[2\left({\left(\frac{\partial U}{\partial X}\right)}^{2}+{\left(\frac{\partial V}{\partial Y}\right)}^{2}\right)+{\left(\frac{\partial^{2}U}{\partial Y^{2}}+\frac{\partial^{2}V}{\partial X^{2}}\right)}^{2}\right]+N_{\mu }\frac{\sigma_{nf}}{\sigma_{f}}Ha^{2}V^{2}$$

where

$$N_{\mu }=\frac{\mu_{f}T_{0}}{k_{f}}{\left(\frac{\alpha_{f}}{L\left(\Delta T\right)}\right)}^{2}$$

Denotes the irreversibility distribution ratio and $S_{GEN}=S_{gen}\frac{T_{0}^{2}L^{2}}{k_{f}{\left(\Delta T\right)}^{2}}\;.\;$ The terms of Equation (27) can be separated into the following form:

(24) $$S_{GEN}=S_{\theta }+S_{\psi }+S_{B}$$

where $S_{\theta }$ denotes the entropy production resulting from heat transfer irreversibility (HTI), $S_{\psi ,}$ denotes the entropy production resulting from fluid friction irreversibility (FFI), and $S_{B}$ denotes the entropy production resulting from magnetic field impacts:

(25) $$S_{\theta }=\frac{k_{nf}}{k_{f}}\left[{\left(\frac{\partial \theta }{\partial X}\right)}^{2}+{\left(\frac{\partial \theta }{\partial Y}\right)}^{2}\right]$$

(26) $$S_{\psi }=\frac{\mu_{nf}}{\mu_{f}}N_{\mu }\left[2\left({\left(\frac{\partial U}{\partial X}\right)}^{2}+{\left(\frac{\partial V}{\partial Y}\right)}^{2}\right)+{\left(\frac{\partial^{2}U}{\partial Y^{2}}+\frac{\partial^{2}V}{\partial X^{2}}\right)}^{2}\right]$$

(27) $$S_{B}=N_{\mu }\frac{\sigma_{nf}}{\sigma_{f}}Ha^{2}V^{2}$$

The Bejan number reads as:

(28) $$B_{e}=\frac{\int S_{\theta }\mathrm{dXdY}}{\int S_{GEN}\mathrm{dXdY}}$$

In fact, the equations for the thermal proprieties were adopted based on the foregoing works, such as [42] and [46].

## 4. Method of Solution

The dimensionless governing equations of Equations (12)–(15) regulated by the assumed boundary conditions of Equations (16)–(20) are solved by the Galerkin weighted and finite element resolution techniques. The adopted Galerkin weighted residual method is employed to handle the governing equations into a constitution of integral mathematical equations. The first step is the discretization process of the computational domain into small triangular elements, as illustrated in Figure 1. Lagrange triangular finite elements of multiple shapes are applied to each concerning flow parameters inside the computational region. The residuals for any conservation equation are reached by replacing the approximations of the dimensionless governing equations. To explain the nonlinear expressions in momentum equations, the Newton–Raphson iteration algorithm is employed. Convergence concerning the solution is only adequate if the following convergence criteria for the relative error of each variable are achieved:

$$\left|\frac{\Gamma^{i+1}-\Gamma^{i}}{\Gamma^{i+1}}\right|\leq \eta$$

where *i* indicates the iteration value and *η* represents the convergence criterion. In this numerical study, the convergence criterion was defined as *η*; = 10^−6^.

### Validation and Grid Independence

**Figure 2:**
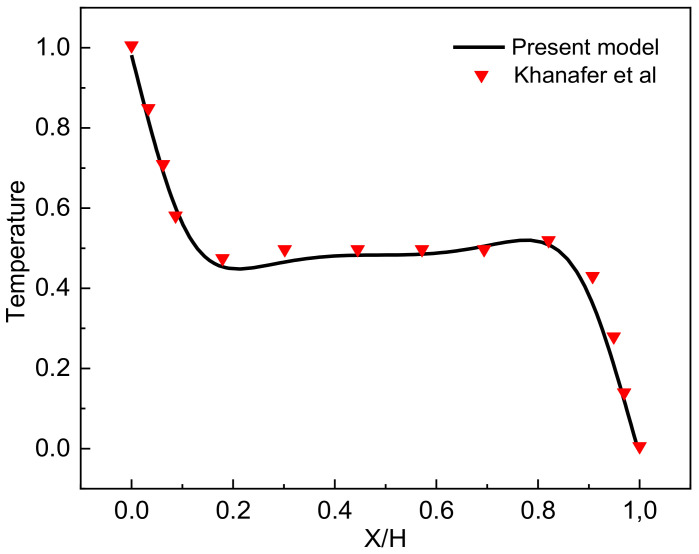
Dimensionless Temperature Distribution for (Ra = 10^5^ and Pr = 0.7) [57]. Reprinted with permission from Elsevier (2003). Copyright 2003.

Grid independency is checked by testing the effect of the mesh size on the average Nusselt number for several mesh configurations (Table 2), and a grid size of 40,600 is selected. To assure the accuracy of the numerical method of the adopted code, the flow behavior is represented by streamline, the thermal behavior is represented by an isotherm that is compared with the former numerical research published by Khanafer et al. [57]. Figure 2 shows the comparison between the results for the profile dimensionless temperature. Comparing the results, it is noted that there is a great agreement between them. Accordingly, it can be said that the method adopted in this work is very accurate.

## 5. Results and Discussion

We mention here that the studied physical field consists of a tightly closed room filled with water and a small percentage of particles of Al_2_O_3_-Cu (nanoparticles) of 4%. The upper wall moves at a constant speed and has a low temperature, while the lower wall has a high temperature and wavy form. An obstacle is inserted in the middle of the room. This investigation aims to determine the effect of some pertinent factors on the heat transfer between the hot wall and the cold one.

Remember also that the fluid inside the container moves due to the following effects: the horizontal movements of the wall move with it the neighboring fluid layers, and then the movement gradually moves between the neighboring fluid layers due to the adhesion resulting from the viscosity of the fluid; the second factor is that cold fluid spots become heavy due to their condensation and this causes them to migrate downwards, while hot spots are lighter and this allows them to move upwards. This successive transition of hot and cold spots produces a free flow. When the fluid motion due to the first factor combines with the second, they form a mixed motion. Applying this mixed motion, the heat transfer is called mixed convection. 

**Figure 3:**
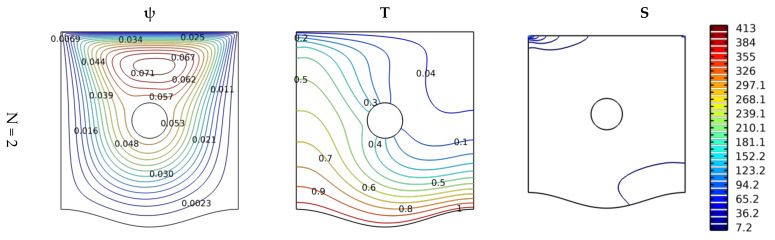

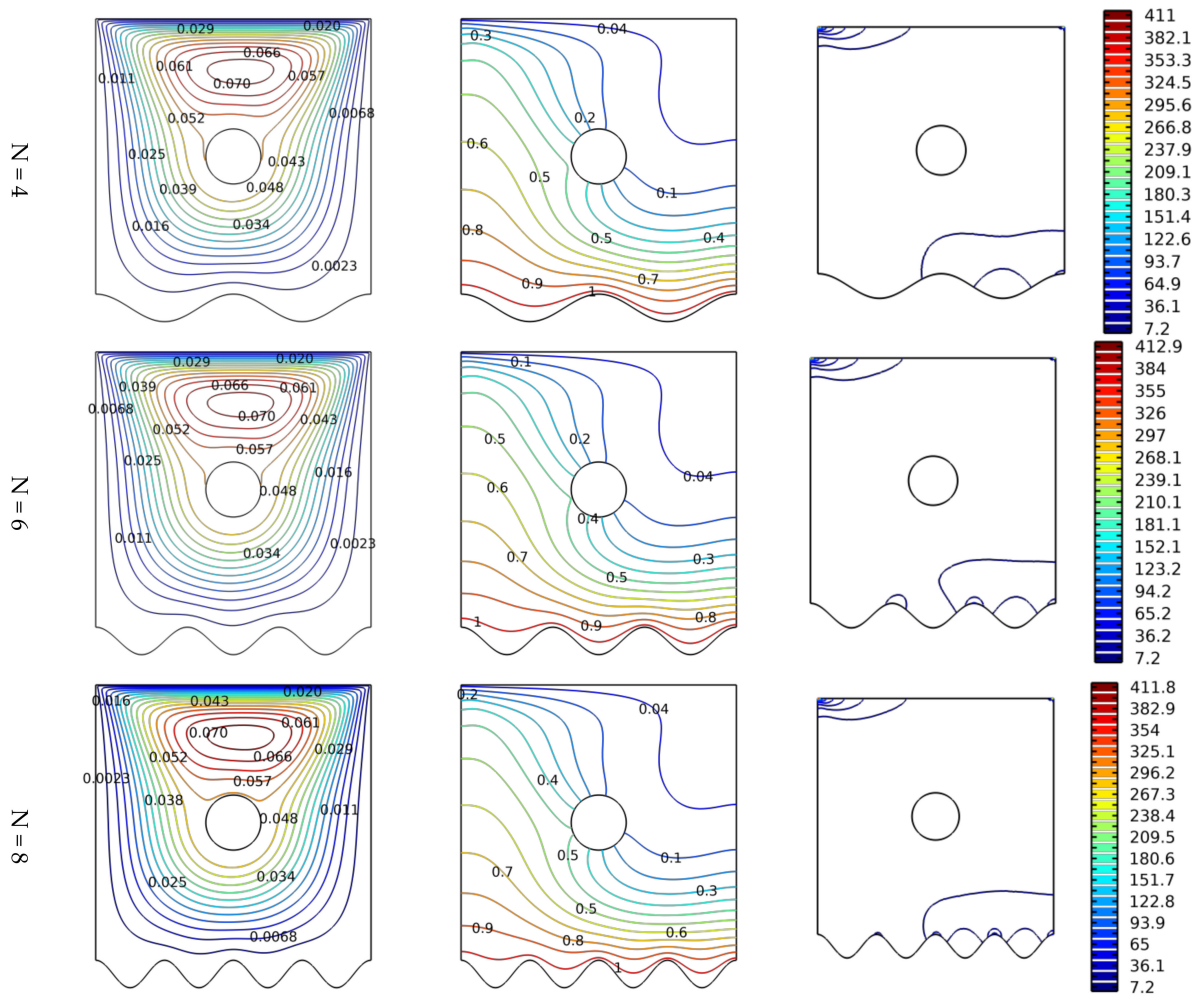
Distribution of streamlines, isotherms, and isentropic contours for various undulation numbers N at Gr = 10^3^, Ha = 0, and Da = 10^−2^.

Figure 3 reflects the nanofluid’s thermal, dynamic and energetic behaviors in terms of the change in the lower wall wave number for Gr = 10^3^, Ha = 0, and Da = 10^−2^. The cross-section in this case of the obstacle is circular in shape. Through the fluid motion contours (streamlines), it is shown that there is a circular flow arising inside the chamber. The center of this circular motion is just above the baffle. It can be seen that the high wall wave number leads to the obstruction of the flow on the lower side, and this reduces its velocity. This effect causes a decline in a thermal distribution near the bottom wall, i.e., we find a decrease in the temperature gradient in terms of the increase in the wave number, and, i.e., we can conclude here that an augmentation in the wave number reduces thermal activity. This analysis can also be verified by the entropy generation values that appear on the upper side of the container on the right and on the lower side on the left. That is, in these areas, the movement of the fluid elements is very important; this makes the energy distribution here important. Through Figure 3, it is clear that the wave shape of the bottom of the chamber makes the movement of the nanofluid particles more difficult. Therefore, a decrease in flow velocity is observed, which decreases both heat transfer and entropy generation.

**Figure 4:**
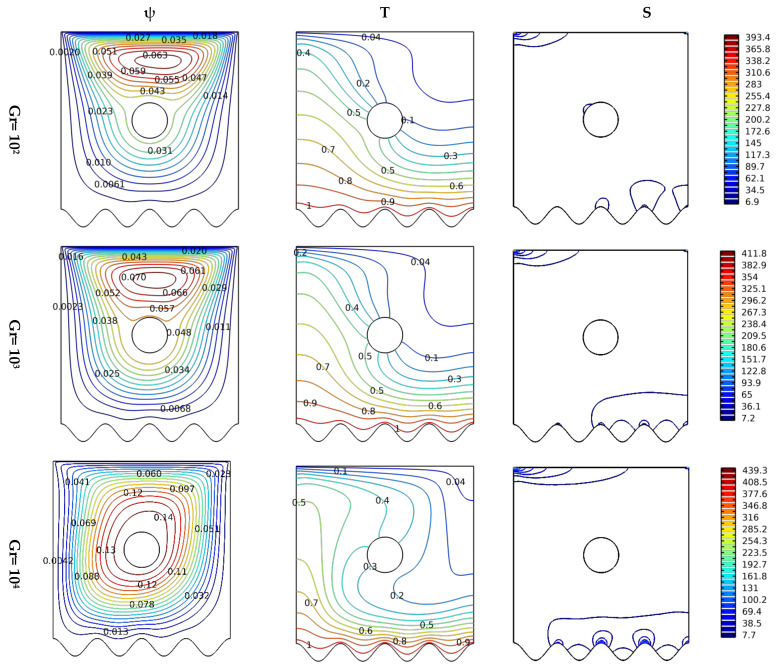
Distribution of streamlines, isotherms, and isentropic contours for various Gr numbers at N = 8, Ha = 0, and Da = 10^−2^.

Figure 4 explains the effect of thermal buoyancy strength on flow movement and its retroactive influence on thermal transfer. Therefore, the streamlines, isotherms, and isentropics are represented in terms of Gr (10^2^, 10^3^, and 10^4^) for Ha = 0, Da = 10^−2^, and N = 8. The circular form is selected for the insulated obstacle. Figure 4 enables us to conclude that the greater the value of Gr, the greater the intensity of buoyancy impact, and this is what makes the velocity of the flow important in the space. Also, the velocity of the flow leads to the deviation of the center of the rotation of the flow downwards. As for the thermal pattern (isotherms), an increase in Gr value increases the temperature gradient near the bottom side of the container, reflecting positivity on the thermal transfer process between the wavy wall and the fluid elements. As for the contours of isentropic, the expansion of the total entropy diffusion in terms of Gr values is observed, and this is, of course, a result of the increase in the fluid velocity due to the force of buoyancy. It can be summarized that increasing the value of Gr increases the density of the cold spots and increases the dilation of the hot spots of the fluid, which makes them move rapidly. It can be concluded that simply raising the value of the Gr number increases the effect of buoyancy force and, therefore, an increase in the speed of the nanofluid particles’ transfer, and this is what makes the thermal transfer and entropy generation more effective. 

**Figure 5:**
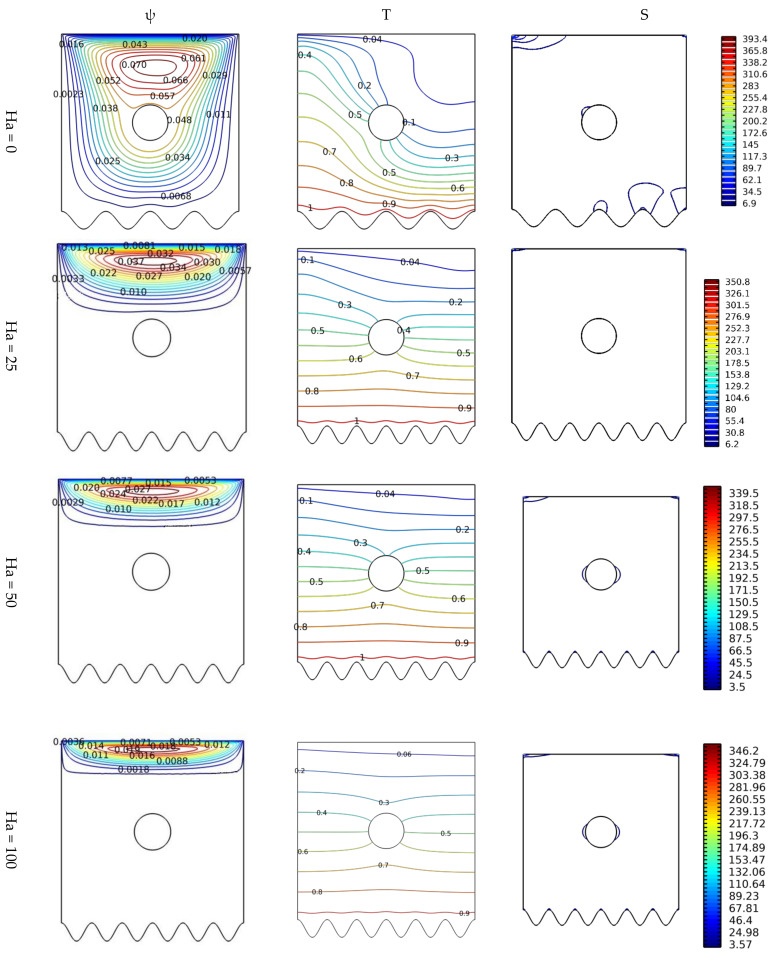
Distribution of streamlines, isotherms, and isentropic contours for various Hartmann numbers Ha at Gr = 10^3^, N = 8, and Da = 10^−2^.

Figure 5 depicts the effect of the increase in the intensity of the magnetic field on the movement of the nanofluid particles, thermal diffusion, and the distribution of entropy generation. The presence of a magnetic field produces a Lorentz force that acts opposite to the buoyant force. Therefore, the appropriate contours are, respectively, shown in terms of Ha number for N = 8, Gr = 10^3^, and Da = 10^−2^; the cross-section always remains circular. Therefore, we note that the flow movement is concentrated only in the upper face near the moving wall. Also, the greater the value of Ha, the greater the contraction in the size of the circular region of the flow. This decrease in the flow speed, especially near the hot wall, resulted in a decrease in the resulting temperature gradient and total entropy generation, as shown in the appropriate elements of Figure 5.

**Figure 6:**
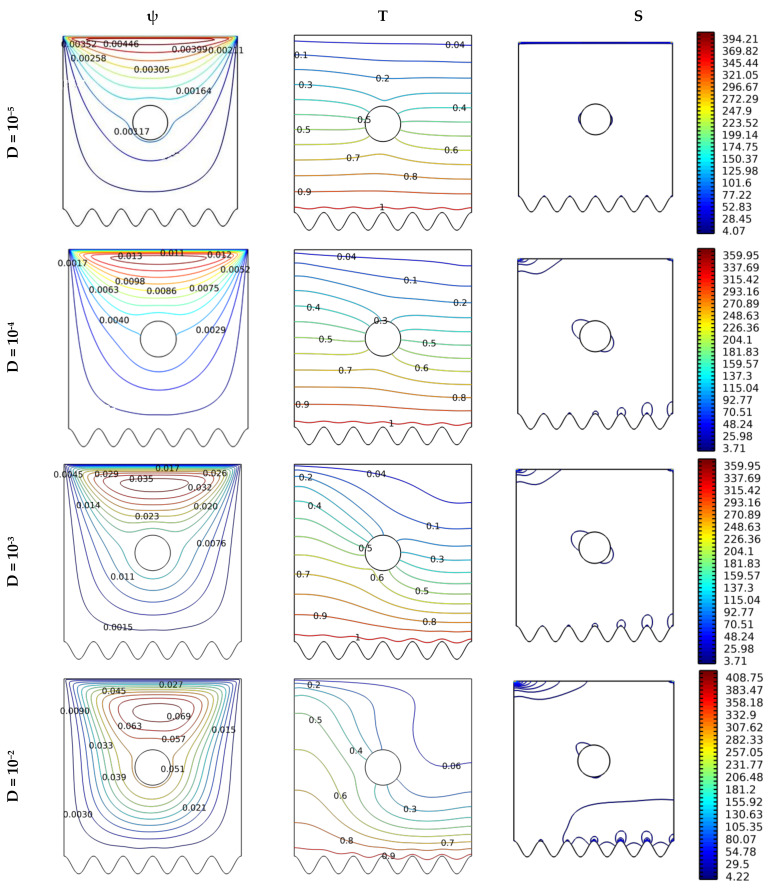
Distribution of streamlines, isotherms, and isentropic contours for various Darcy numbers Da at Gr = 10^3^, N = 8, and Ha = 0.

Figure 6 describes the effect of the medium permeability on thermal activity and the dynamic model of the motion of the fluid particles. It is known that raising the Da number increases the permeability of the space and accordingly decreases the resistance of the medium to the flow of the fluid, which makes the fluid velocity better. So the streamlines are depicted in terms of Darcy number for N = 8, Gr = 103, and Ha = 0, and for a circular form. Based on this, an increase in temperature gradient is observed near the hot wall, which reflects positively on the relationship between Darcy numbers (permeability of the space) and thermal activity.

**Figure 7:**
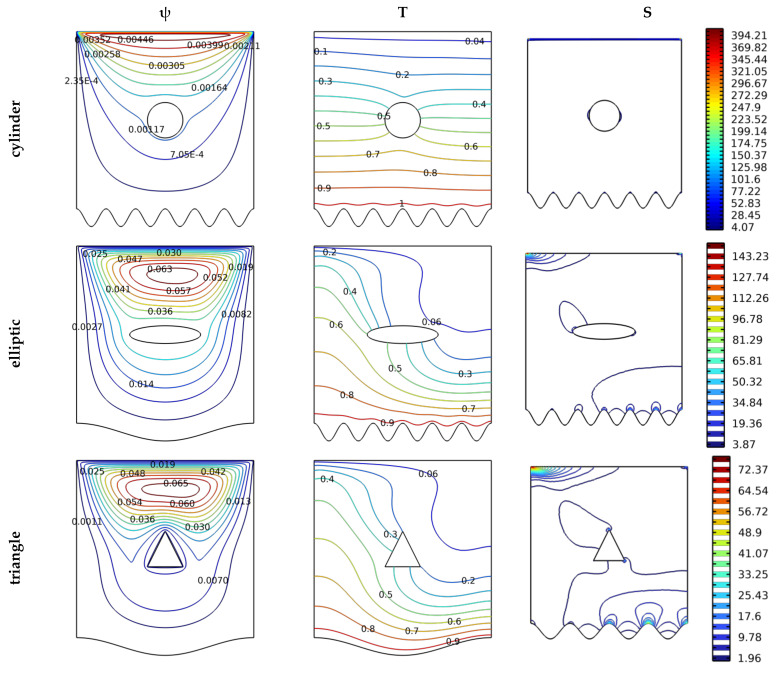

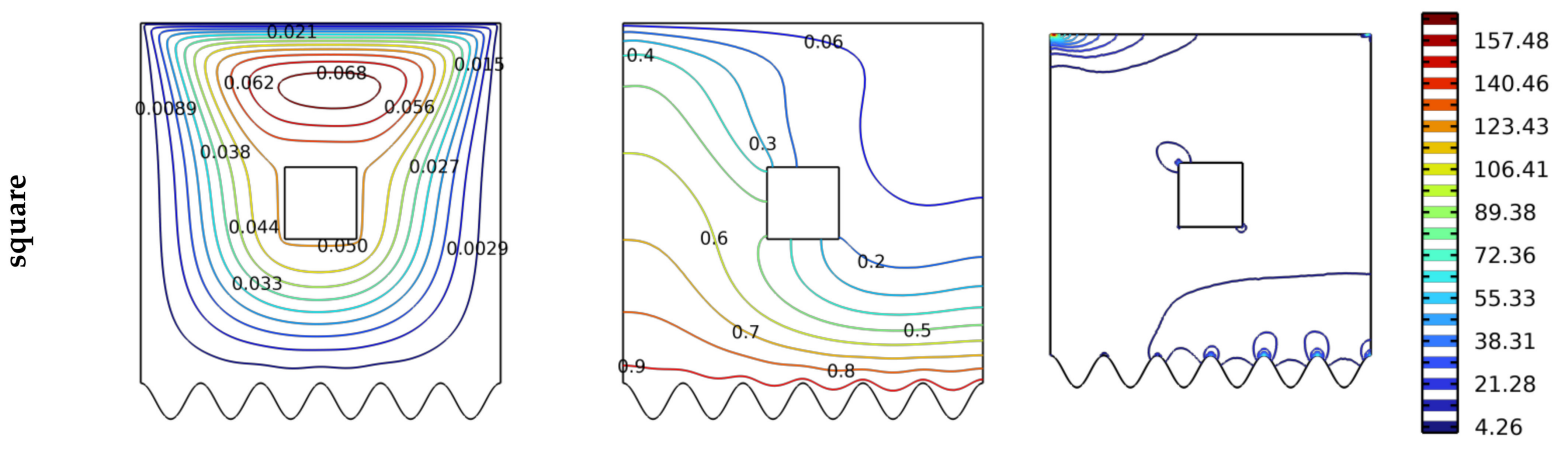
Distribution of streamlines, isotherms, and isentropic contours for various cases Gr = 10^3^, N = 8, and Ha = 0.

The baffles’ form is very important in directing the flow, which hinders its progress or helps it. Figure 7 shows the effect of the baffle’s cross-sectional shape (circular, elliptical, triangular, or square) on the thermal and dynamic comportments of the hybrid nanofluid. The effect of this change is made for the following values Da at Gr = 103, N = 8, and Ha = 0. From Figure 7, we note that the path of the fluid is affected by the shape of the cross-section of the baffle. The flow velocity is considered for the triangular and the square shapes. Also, the temperature gradient near the hot wall is considered for the triangular shape and, then, the square shape. The triangular shape allows for more quantity generation of entropy, followed by the square shape. The same goes for the entropy generation. 

**Figure 8:**
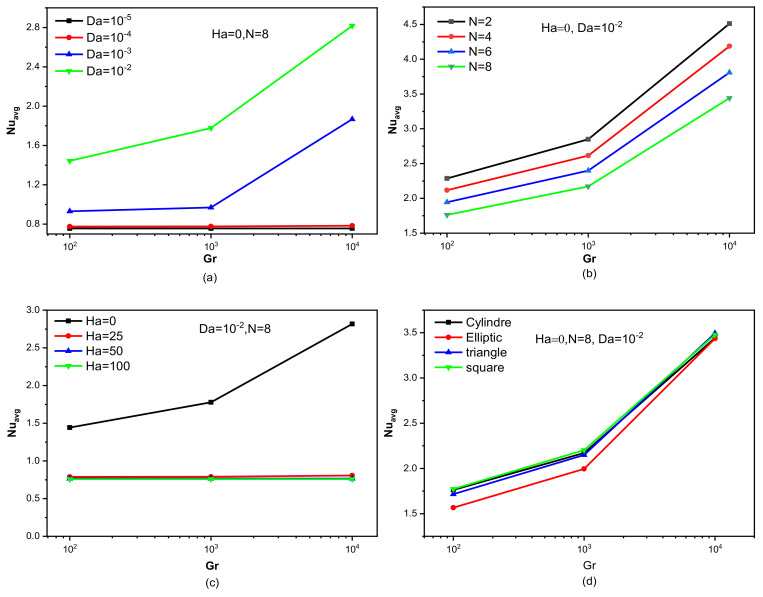
Average Nusselt number versus Grashof numbers for different (**a**) Darcy numbers, (**b**) undulation numbers, (**c**) Hartmann numbers, and (**d**) obstacles shapes for Da = 10^−2^, Ha = 0, and N = 8.

Figure 8 represents the developments in the mean values of Nu number of the hot wall in terms of the previously studied and mentioned elements. In fact, the Nusselt number defines the ratio between the heat transfer caused by the movement of the fluid’s particles (convection) and the heat transfer that the medium allows to pass through (conduction). Figure 8a describes the development of the *Nu* number in terms of Gr and Da for Ha = 0 and N = 8, and the circular form of the obstacle. It is clearly noticed that the higher value of Da and/or Gr leads to an increase in the value of Nu, and this is, of course, a normal thing; the increase in the permeability of the medium reduces the obstruction of the flow movement, which keeps the velocity better, and this makes the heat transfer better. On the other hand, raising the value of Gr makes the flow move faster, and this enhances the heat activity. The effect of the wave number on Nu of the heated wall is depicted in Figure 8b for Ha = 0 and Da = 10^−2^, and the cross-section is circular. It becomes clear that the more wavy the wall is, the lower the value of the Nu number, because this shape of the wall does not allow good friction between the flow and the surface of the wall, which reduces the thermal transfer, and, therefore, we find a decrease in the Nu number. The effect of magnetic field strength on Nu is represented in Figure 8c for Da = 10^−2^ and N = 8, and the obstacle is also circular. As expected, increasing the value of Ha negatively affects Nu. This is because of Lorentz’s force that hinders the nanofluid flow, which slows down heat activity and, therefore, leads to a decrease in the value of Nu. Figure 8d reflects the impacts of the obstacle cross-section on Nu values for Ha = 0, Da = 10^−2^, and N = 8. It is noted in Figure 8d that there is a clear change in the values of the Nu in terms of the shape of the cross-section. It is noted that the triangular and the square shapes are the best in terms of thermal activity.

**Figure 9:**
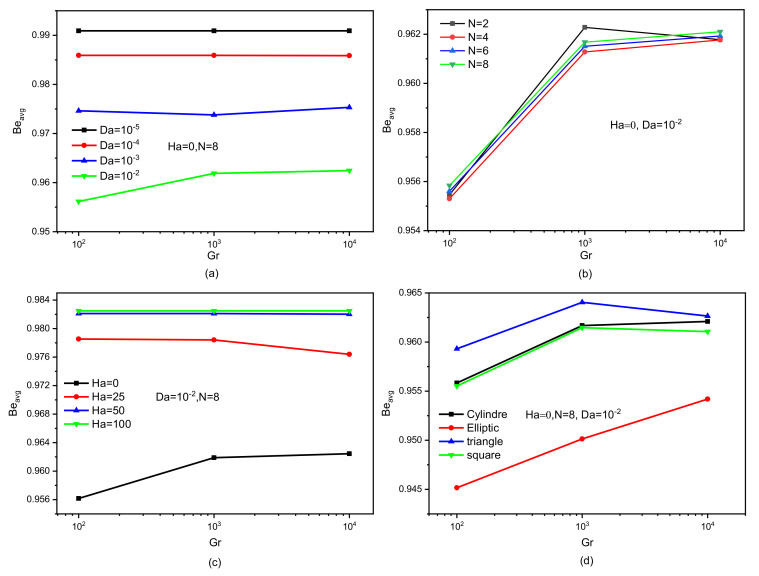
Bejan number versus Grashof numbers for different (**a**) Darcy numbers, (**b**) undulation numbers, (**c**) Hartmann numbers, and (**d**) obstacle shapes for Da = 10^−2^, Ha = 0, and N = 8.

Figure 9 shows the influences of the studied elements on the values of the Bejan number. This number expresses the ratio of entropy formed due to thermal source over the entropy resulting from the friction of the fluid layers. From all the charts in Figure 9, it is clear that the entropy generated due to thermal activity is dominant in this work. Figure 9a shows the influence of Da for Ha = 0 and N = 8. Since the permeability of the space increases in terms of Da, the value of Be decreases because the movement of the flow increases the friction. Figure 9b represents the variation of Be in terms of the wave number for Ha = 0 and Da = 10^−2^. Of course, the greater the wave number, the less the flow’s movement, which causes the values of Be to increase. Figure 9c is intended to show the effect of Ha on Nu for Da = 10^−2^ and N = 8. It was found previously that the increase in the value of Ha negatively affects the speed of the flow, and this is what makes the thermal effect on entropy generation. Therefore, we note that the higher the Ha peak, the more important is the Be value. Figure 9d shows the effect of the cross-sectional shape of the baffle on Be for Ha = 0, N = 8, and Da = 10^−2^. The effect of the cross-section of the baffle on the Be value is actually observed due to the shape of the obstacle that changes the kinetic path of the flow.

## 6. Conclusions

This research deals with a numerical study of nanofluid inside the lid-driven chamber. The nanofluid is a hybrid type that contains 4% of Al_2_O_3_-Cu nanoparticles. The container also includes a thermally insulated baffle. The upper wall is adiabatic and horizontally movable, and the lateral sides have low temperatures, while the lower wall is hot and contains waves. This research aims to assess the thermal transfer between the hot and cold walls by the intervention of a group of influential elements, namely: the Darcy number, the Hartmann number, the Grashof number, the wave number, and, finally, the form of the cross-section of the obstacle. The results of this work enable us to derive the following points:

- An increase in wall wave number reduces the thermal activity of the hot wall;
- Increasing the permeability of the medium allows fluid to flow, making heat transfer more efficient;
- The presence of the magnetic field induces the Lorentz force, which impedes the movement of the fluid, resulting in a decrease in thermal activity;
- The velocity of the fluid movement inside the chamber increases as the thermal buoyancy increases, i.e., an increase in the heat-transfer coefficient;
- Triangular and square shapes of the baffle are the best for improving heat transfer;
- At the highest Gr number, increasing the wall wave number and the Ha number reduced Nu by 28% and 73%, respectively—while increasing the Da number increased Nu by 260%;
- The use of the elliptical shape of the inner cylinder is very effective in the case of thermal insulation; and
- The change in the shape of the obstacle from elliptical to triangular raises the value of the Nu number at a rate of 15.54% for Ha = 0, N = 8, and Gr = 10^4^.
- Regarding future works, the following can be suggested:
- Raising the value of the Reynolds number and studying phenomena in terms of time;
- Using a complex fluid as the base fluid instead of water; and
- Suggest other forms of the obstacles.

The new findings in this work compared to previous works that this work shows the best forms of the obstacle that will enhance the heat transfer.

## Figures and Tables

**Table 1:** Thermophysical characteristics at 293 K [41] and [46].

Material	ρ $\left(\mathrm{kg}/m^{3}\right)$	$C_{p}$ (J/kg k)	$\mu \times 10^{6}$ (Pa s)	$\beta \times 10^{5}$ (1/k)	k (W/m k)	σ (S/m)
Alumina (Al_2_O_3_)	3970	765	-	0.85	25	10^−10^
Water	997.1	4179	695	21	0.613	0.05

**Table 2:** Grid sensitivity check for the case 5 (Ha = 0, φ = 0.04, Da = 10^−2^, and Ra = 10^5^).

Number of Elements	940	1534	2442	10,920	40,600	43,900
CPU Time (s)	30	45	50	66	72	120
Nu_avg_	8.8197	9.1230	9.3964	10.299	11.207	11.200
$\psi_{\max}$	2.4309	2.4309	2.4437	2.4504	2.451	2.4520

## Data Availability

Not applicable.

## Nomenclature

B_0_	magnetic induction (Wb/m^2^)
Be	Bejan number
C_p_	specific heat at constant pressure (J/kg K)
g	gravity (m^2^/s)
Gr	Grashof number
Ha	Hartman number
k	thermal conductivity of air (W/m K)
L	dimension of the cavity (m)
N	undulation
Nu	average Nusselt number
p	pressure (Pa)
P	non-dimensional pressure
Pr	Prandtl number
Re	Reynolds number
Ri	Richardson number
S	entropy (J/K)
T	temperature (K)
U_o_	moving lid velocity (m/s)
u, v	velocity components (m/s)
U, V	non-dimensional velocity components
x, y	coordinates (m)
X, Y	non-dimensional coordinate
Greek Symbols	
α	thermal diffusivity (m^2^/s)
*β*	thermal expansion coefficient (1/K)
γ	rotation angle (deg)
*φ*	nanoparticles volume fraction
*μ*	dynamic viscosity (kg m/s)
*θ*	dimensionless temperature
*ρ*	density of fluid (kg/m^3^)
*σ*	fluid electrical conductivity (S/m)
*ψ*	irreversibly
Subscripts	
Gen	generation
nb	base fluid
nf	nanofluid
avg	average
c	cold
h	hot
s	surface
